# Orally deliverable strategy based on microalgal biomass for intestinal disease treatment

**DOI:** 10.1126/sciadv.abi9265

**Published:** 2021-11-24

**Authors:** Danni Zhong, Dongxiao Zhang, Wei Chen, Jian He, Chaojie Ren, Xingcai Zhang, Na Kong, Wei Tao, Min Zhou

**Affiliations:** 1 Eye Center, the Second Affiliated Hospital, Zhejiang University School of Medicine, Hangzhou, China.; 2 Institute of Translational Medicine, Zhejiang University, Hangzhou, China.; 3 Center for Nanomedicine and Department of Anesthesiology, Brigham and Women’s Hospital, Harvard Medical School, Boston, MA 02115, USA.; 4 School of Engineering and Applied Sciences, Harvard University, Cambridge, MA 02138, USA.; 5 State Key Laboratory of Modern Optical Instrumentations, Zhejiang University, Hangzhou 310058, China.

## Abstract

Design of innovative strategies for oral drug delivery is particularly promising for intestinal disease treatment. However, many obstacles such as poor therapeutic efficacy and low bioavailability and biocompatibility remain to be addressed. Here, we report a versatile formulation based on a helical-shaped cyanobacterium, *Spirulina platensis* (SP), loaded with curcumin (SP@Curcumin) for the treatment of colon cancer and colitis, two types of intestinal diseases. In radiotherapy for colon cancer, SP@Curcumin could mediate combined chemo- and radiotherapy to inhibit tumor progression while acting as a radioprotector to scavenge reactive oxygen species induced by the high dose of x-ray radiation in healthy tissues. SP@Curcumin could also reduce the production of proinflammatory cytokines and thereby exerted anti-inflammatory effects against colitis. The oral drug delivery system not only leveraged the biological properties of microalgal carriers to improve the bioavailability of loaded drugs but also performed excellent antitumor and anti-inflammation efficacy for intestinal disease treatment.

## Teaser

Microalgae-based drug delivery system exhibits efficient therapeutic effects on intestinal diseases.

## INTRODUCTION

Oral drug delivery is the preferred and most commonly used route of drug administration for gastrointestinal (GI) disease treatment, mainly owing to its safety, high patient compliance, convenience, and ease of production (*1*–*4*). However, numerous challenges remain in oral drug delivery, such as the degradation of active pharmaceutical ingredients in the acidic environment of stomach, which results in their poor retention and low bioavailability in the intestine (*5*–*8*). To improve the efficacy of oral administration, various drug carriers have been applied to the design of oral delivery systems, such as liposomes (*9*–*11*), dendrimers (*12*, *13*), micelles (*14*), polymer conjugates (*15*), polymeric nanoparticles (*16*, *17*), silicon or carbon materials (*18*, *19*), and metal and magnetic nanoparticles (*20*, *21*). Generally, the multifunctionality of such candidates is achieved through the complex design, synthesis, and construction processes to optimize their physicochemical properties, thus allowing their use in drug delivery and theranostic applications. Yet, major concerns remain in the technical challenges, high manufacturing costs, and low effectiveness resulting from such complicated synthetic processes (*22*, *23*). The feasibility of translating these chemically synthesized and engineered materials into clinical use is severely limited by their low biodegradability, undesirable stability, and potential toxicity in vivo (*24*–*26*). Therefore, it is highly desirable to develop a facile, versatile, and biocompatible strategy for oral drug delivery.

Microalgae, which are abundant, natural, and renewable biological resources, have recently attracted substantial attention in biomedical applications. Microalgae can be cultivated in large quantities, and many species of them have been commercialized as nutritional and food supplements, proving their practicability and high biosafety as oral pharmaceutical formulations (*27*, *28*). Furthermore, many studies have demonstrated the strong potential of microalgae for targeted drug delivery both in vitro and in vivo, as they can effectively load drug molecules through their active surfaces (*29*–*31*). Given this paradigm, we propose to use the helical-shaped cyanobacterium, *Spirulina platensis* (SP), as a possible drug carrier for oral drug delivery. The spiral structure of SP may enable it to be not only more easily trapped by the intestinal villi but also adhered to the intestinal wall, thereby prolonging the retention time of the drugs in the intestine. Furthermore, the intrinsic fluorescent chlorophyll produced by SP allows to perform the noninvasive fluorescence imaging without the need for chemical modification (*32*, *33*), rendering the multifunctional SP particularly suitable for both therapeutic and diagnostic applications. In this sense, the utilization of microalgal biomass–based drug delivery system may offer innovative means for reducing costs, minimizing toxicity, and improving therapeutic efficacy of oral administration.

**Fig. 1.**
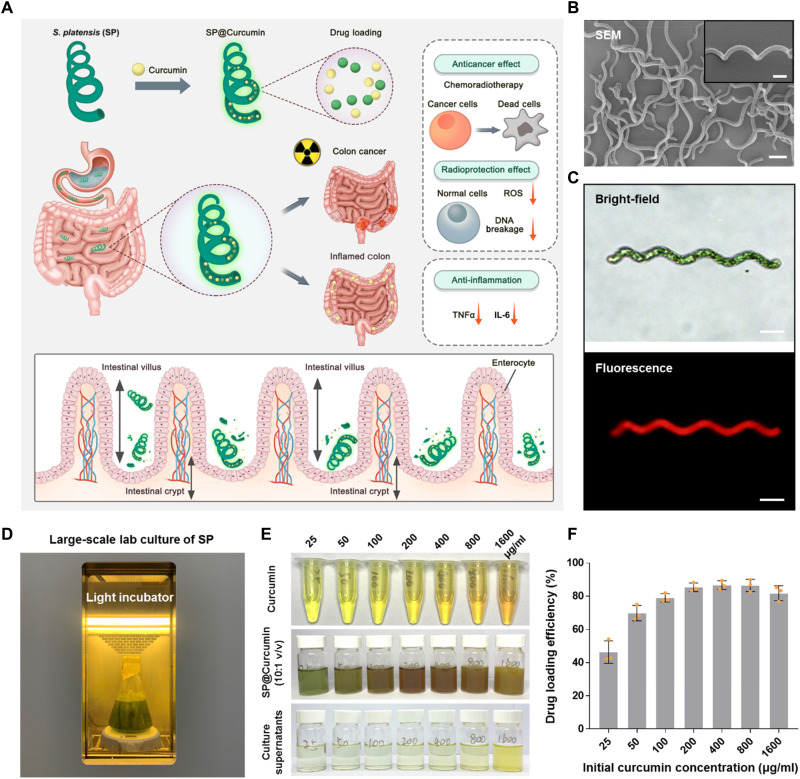
Characterization of the SP@Curcumin system. (**A**) Schematic illustration of SP@Curcumin-mediated (i) chemoradiotherapy of colon cancer, including the inhibition of tumor cells and the radioprotection of normal cells, and (ii) anti-inflammatory effects against intestinal inflammation. (**B**) SEM image of SP. Scale bar, 20 μm. (Inset: high-resolution SEM image of SP; scale bar, 10 μm.) (**C**) Bright-field and fluorescence images of SP. Scale bars, 20 μm. (**D**) Photograph of the large-scale laboratory culture of SP. (**E**) Photograph of the drug loading process of equivalent SP mixing with curcumin at different concentrations. (**F**) Drug loading efficiency of SP at a series of initial loading concentrations. Photo credit: Danni Zhong, Zhejiang University.

Here, we used SP as a carrier to construct a microalgae-based oral drug delivery system (SP@Curcumin) for the treatment of multiple intestinal diseases (Fig. 1A). Curcumin, a U.S. Food and Drug Administration–approved drug, which has a variety of pharmacological functions such as anti-inflammation and anticancer (*34*–*36*), was loaded in SP. We demonstrated that SP@Curcumin could pass through the stomach while its structure remained intact. Then, SP@Curcumin could be captured by the intestinal villi and gradually degrade and release curcumin, thus achieving a desirable drug distribution in the intestine without inducing adverse effects (movie S1). In traditional radiotherapy for colon cancer (i.e., the first intestinal disease model), SP@Curcumin exhibited synergistic therapeutic effects by combining chemotherapy and radiotherapy to inhibit tumor progression. Meanwhile, SP@Curcumin also protected normal intestinal tissue during radiotherapy by eliminating reactive oxygen species (ROS) production in normal cells and reducing ROS-induced DNA damage. In addition to its application in cancer therapy, we also demonstrated the anti-inflammatory ability of SP@Curcumin in the intestine, which reduced the level of proinflammatory cytokines and diminished the inflammation-associated symptoms of mice with colitis (i.e., the second intestinal disease model). This work presents a multifunctional drug delivery system that can bypass the physiological barriers, improve the pharmaceutical properties (e.g., oral bioavailability, biodegradation, and biocompatibility), and achieve the combination therapy and theranostics.

## RESULTS

### Characterization and drug loading performance of SP

In this study, we used natural SP with a standard helical shape as a drug carrier whose length and width are approximately 100 and 5 μm, respectively (Fig. 1B). SP is green in color. Because of the abundant chlorophyll in its structure, SP emits strong red fluorescence when exposed to appropriate excitation light (~605 nm) (Fig. 1C). Besides, the large-scale culture of SP could be easily carried out in the laboratory setting (Fig. 1D). To evaluate the drug loading ability of SP, various concentrations of curcumin (1 ml; 25, 50, 100, 200, 400, 800, and 1600 μg/ml) were added to SP samples (10 ml, at the equivalent SP concentration of 50 μg/ml). The drug loading process was a single step, lasting for 12 hours at room temperature, and finally the as-prepared SP@Curcumin was collected by centrifugation. When the initial concentration of curcumin was less than 800 μg/ml, there was no obvious yellow color in the supernatants of the mixed cultures, indicating that SP could effectively load curcumin (Fig. 1E). The drug loading efficiencies at different initial drug concentrations were calculated using the standard curve of the curcumin solutions (fig. S1), in combination with the ultraviolet-visible (UV-vis) absorptions of the culture supernatants (fig. S2). As shown in Fig. 1F, when the initial curcumin concentration was higher than 200 μg/ml, the drug loading efficiency was more than 80%. The drug loading efficiency reached a maximum when the concentration of curcumin was 400 μg/ml, which yielded the optimal drug/carrier ratio for the preparation of SP@Curcumin for the subsequent experiments. The structural stability of SP@Curcumin was demonstrated as no notable changes in the morphology of SP@Curcumin were observed during the 2-week storage in deionized (DI) water at room temperature (fig. S3). In addition, the as-prepared SP@Curcumin suspension could not only be made into freeze-dried powder for long-term storage but also be easily resuspended in DI water, which facilitates the application and commercialization of SP@Curcumin formulations (fig. S4).

### Fluorescence imaging and distribution of SP@Curcumin

**Fig. 2.**
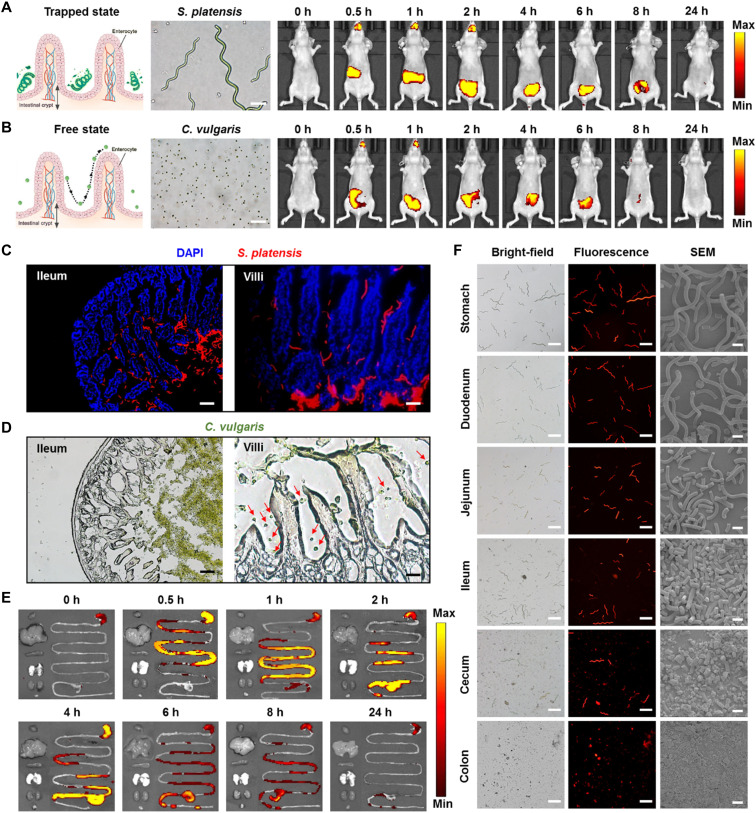
In vivo fluorescence imaging and biodistribution. Time-dependent in vivo fluorescence images of Balb/c nude mice after the intragastrical administration with (**A**) large spiral-shaped SP or (**B**) small spherical *C. vulgaris*. Scale bars, 20 μm. (**C**) Frozen slices of ileum (left) and ileal villi (right) stained with 4′,6-diamidino-2-phenylindole (DAPI) (blue, nuclei). The red signals are SP. The scale bars are 100 and 50 μm for ileum and villi, respectively. (**D**) Bright-field images of frozen slices of ileum (left) and ileal villi (right). The red arrows are *C. vulgaris*. The scale bars are 100 and 25 μm for ileum and villi, respectively. (**E**) Ex vivo fluorescence images of the major organs/tissues, including heart, liver, spleen, lung, kidney, bladder, and GI tract of mice collected at different time points after the intragastrical administration with SP@Curcumin. (**F**) Bright-field, fluorescence, and SEM images of the contents in different parts of the GI tract, showing the morphological changes and degradation of SP@Curcumin after the intragastrical administration. The scale bars are 100, 100, and 10 μm, respectively.

Because the chlorophyll in SP is capable of emitting red fluorescence, we investigated whether this inherent feature could be applied to perform the in vivo fluorescence imaging and to noninvasively evaluate the biodistribution of SP@Curcumin. First, we performed in vitro fluorescence imaging of SP and SP@Curcumin. Both of them exhibited good fluorescence imaging performance under the selected channel (excitation, 605 nm; emission, 615 to 665 nm; Cy5.5), and the quantitative analysis of fluorescence signals showed no statistically significant difference, indicating that drug loading had no obvious effect on the fluorescent properties of SP (fig. S5). Next, we investigated the distribution of SP in the GI tract of mice after intragastrical administration. As shown in Fig. 2A, the fluorescence signal was first located predominantly on the upper side of the abdomen and gradually moved downward, indicating that SP passed through the stomach and intestines. Similar results were observed in SP@Curcumin-treated mice, suggesting that the loaded drug did not affect the fluorescence ability and distribution behavior of SP in vivo (fig. S6). We hypothesized that the helical structure of SP made it easy to be trapped by the intestinal villi and held close to the intestinal mucosa, thus making the drug delivery system stay in the intestine for an extended period of time. To test this hypothesis, we used a spherical eukaryotic microalga, *Chlorella vulgaris*, and compared its adhesion to intestinal mucosa with that of SP. The fluorescence signal of *C. vulgaris*–treated mice decreased markedly 2 hours after administration, indicating that *C. vulgaris* had been metabolized and excreted from the body, and the fluorescence signal disappeared at 8 hours (Fig. 2B). Conversely, the fluorescence signal of SP-treated mice remained unchanged for 4 hours and was still detectable at 8 hours after administration (Fig. 2A and fig. S6). Furthermore, both bright-field and fluorescence microscope images of frozen tissue sections revealed a large number of SP carriers accumulated in the ileum tissues after intragastrical administration, and many of them were trapped in the villi and adhered to the epithelium (Fig. 2C and fig. S7). Comparatively, few *C. vulgaris* cells could settle in the intestinal epithelium (Fig. 2D). Collectively, these findings indicated that the morphological characteristics of SP contributed to a desirable distribution of this drug delivery system in the intestinal tissues.

To better visualize the distribution of SP@Curcumin in different parts of the GI tract, we performed ex vivo fluorescence imaging on the collected tissues. As depicted in Fig. 2E, at 0.5 hours after intragastrical administration, the fluorescence signal appeared mainly in the stomach and ileum, indicating that SP@Curcumin passed through the stomach quickly. The fluorescent signal was mainly distributed in the ileum before 2 hours, then gradually moved to the cecum and colon, and covered the entire GI tract at 4 hours. After 6 hours, the fluorescence intensity gradually decreased, indicating that SP@Curcumin began to be excreted from the body. During the whole detection period, negligible fluorescence signal was observed in other main organs (heart, liver, spleen, lung, kidney, and bladder), indicating that SP@Curcumin was metabolized in the GI tract after intragastrical administration and spread negligibly to other major organs. To further investigate the degradation process of SP@Curcumin, the contents of different parts in the GI tract (stomach, duodenum, jejunum, ileum, cecum, and colon) were harvested and photographed by optical microscopy and scanning electron microscopy (SEM) (Fig. 2F). The SP carriers in the stomach and duodenum were almost intact as a complete spiral structure was observed. Subsequently, they began to rupture in the jejunum and were completely broken into small pieces in the ileum and cecum. When reaching the colon, they were completely fragmented, showing the biodegradability of SP@Curcumin.

In addition to the good biodegradability, effective protection of encapsulated drugs against the destruction by gastric acid is essential for an excellent drug delivery system. Therefore, we investigated the drug release behavior of SP@Curcumin in simulated gastric fluid (SGF) containing pepsin (pH 2) and in simulated intestinal fluid (SIF) containing trypsin (pH 6.8) to simulate the acidic environment of the stomach (pH ~1.8) and the terminal ileum (pH 6 to 7.4) (*37*), respectively. At 0.5 hours, less than 8% of the drugs was released from SP@Curcumin in the simulated gastric acid environment (fig. S8A), at which time point most of the SP carriers had passed through the stomach (Fig. 2E). Subsequently, ~66% of the drug was released in the simulated intestinal environment within 8 hours (fig. S8B). In addition, no obvious changes in the morphology and length of SP carriers were observed after 120 min of SGF treatment, indicating that SP could effectively resist the degradation by the gastric acid environment (fig. S9). The pharmacokinetic studies showed that the relative abundance of curcumin in mouse plasma was increased significantly and the time point with the maximum relative abundance was prolonged after intragastrical administration of SP@Curcumin when compared to free curcumin treatment (fig. S10). These combined results demonstrated that SP carriers could not only perform noninvasive fluorescence imaging in vivo by fluorescent chlorophyll but also exert protective effects on the loaded curcumin to ensure the sustained drug release in the intestine, thus improving the pharmacokinetic profile of the loaded drug. Last, these SP carriers could be fully degraded in vivo to ensure their biosafety (fig. S11).

### Radiation protection performance

**Fig. 3.**
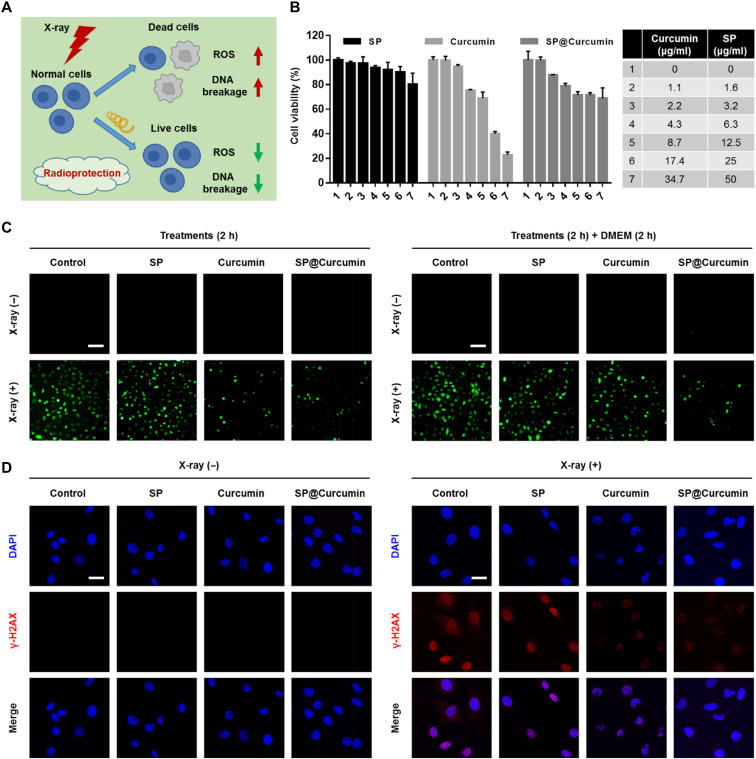
SP@Curcumin-mediated radioprotection of normal intestinal epithelial cells. (**A**) Schematic illustration of SP@Curcumin as a radioprotector. (**B**) Cell viabilities of IEC-6 cells after the incubation with different concentrations of SP, curcumin, and SP@Curcumin for 24 hours. (**C**) Representative fluorescence microscope images of intracellular ROS generation in IEC-6 cells after the different treatments. Top: Cells were incubated with different media for 2 hours before x-ray irradiation (12 Gy). Bottom: After incubating with different media for 2 hours, cells were incubated with fresh DMEM and then irradiated by x-ray after coculture for 2 hours. Scale bars, 100 μm. (**D**) Representative γ-H2AX immunofluorescence images of DNA double-strand damage in IEC-6 cells after the different treatments. Scale bars, 20 μm.

To explore the potential of SP as a drug carrier for further biomedical applications, we first investigated the therapeutic effects of SP@Curcumin on cells. Curcumin has been demonstrated as a free radical scavenger and has been widely applied in radioprotection (*38*). We therefore studied the ability of SP@Curcumin to reduce the radiation-induced ROS generation in normal cells. The results showed that SP@Curcumin reduced the oxidative damage caused by the excess ROS, such as DNA-based modifications by single- and double-strand breaks (Fig. 3A). It is known that small intestine is one of the organs most sensitive to radiation in the human body (*39*). Thus, we chose a normal small intestinal epithelial cell line, IEC-6, to assess the in vitro radioprotective ability of SP@Curcumin. First, the cytotoxicity of SP, curcumin, and SP@Curcumin to IEC-6 cells was evaluated using an MTT [3-(4,5-dimethylthiazol-2-yl)-2,5-diphenyltetrazolium bromide] assay. The cytotoxicity of free curcumin at high concentrations was much higher than that of an equivalent curcumin concentration in the form of SP@Curcumin, indicating the substantially reduced cytotoxicity of SP@Curcumin (Fig. 3B). Then, we examined the ability of SP@Curcumin to reduce ROS production in IEC-6 cells under x-ray radiation. After pretreatment with free curcumin or SP@Curcumin, the green fluorescence intensity of the intracellular ROS induced by x-ray irradiation was significantly lower than that of x-ray treatment only (Fig. 3C and fig. S12). To simulate the adhesion of SP@Curcumin in the intestine, we replaced the cell culture medium containing different drugs by fresh Dulbecco’s modified Eagle’s medium (DMEM) for further coincubation and then irradiated the cells by x-ray. As depicted in Fig. 3C and fig. S12, ROS production in the cells preincubated with free curcumin recovered to a higher level, while the ROS intensity of the cells pretreated with SP@Curcumin remained significantly lower than in other groups. Furthermore, we evaluated the ability of SP@Curcumin to reduce the radiation-induced DNA damage in IEC-6 cells by immunofluorescence staining of γ-H2AX (a marker of double-stranded DNA breakage). After x-ray irradiation, obvious DNA damages in the control and SP-treated groups were observed. However, in the Curcumin + X-ray and SP@Curcumin + X-ray groups, red fluorescent foci were significantly reduced, which showed the reduced ROS expression by curcumin and thus inhibited the interaction between ROS and DNA (Fig. 3D and fig. S13). Moreover, a colony formation assay was performed to evaluate the clonogenic survival of IEC-6 cells under different treatments. Compared with the nonirradiated group, the colony formation of cells in the control and SP-treated groups was significantly lower. In contrast, pretreatment with curcumin or SP@Curcumin before irradiation significantly increased the number of colony formation (fig. S14). Collectively, these in vitro results verified the radioprotection ability of SP@Curcumin to normal cells.

**Fig. 4.**
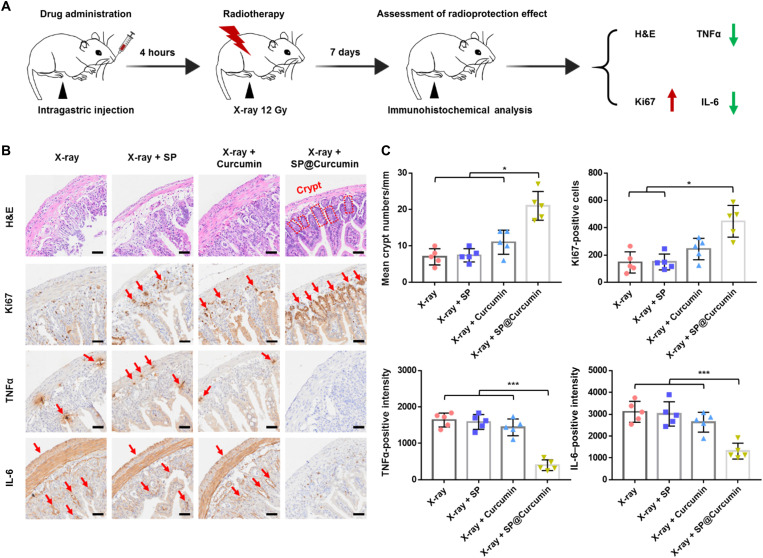
SP@Curcumin-mediated radioprotection of normal intestinal tissues. (**A**) Schematic illustration of SP@Curcumin as a radioprotector against intestinal damage caused by high doses of radiation (12 Gy). (**B**) Representative H&E, Ki67, TNFα, and IL-6 staining images of the ileum tissues in different groups. Scale bars, 50 μm. (**C**) Quantitative analysis of crypt numbers, Ki67-positive cells, and TNFα- and IL-6–positive intensity on 7 days after different treatments. Data are means ± SD; *n* = 5; Student’s two-tailed *t* test, *P < 0.05 and ***P < 0.001.

Encouraged by the favorable radioprotective performance on intestinal cells, we then evaluated the potential of SP@Curcumin for the radioprotection of normal intestinal tissues. According to the results of in vivo biodistribution, mice were given the high-dose radiation of 12 Gy at 4 hours after intragastrical administration, and the small intestines (ileum) were collected 7 days later for pathological analysis (Fig. 4A). As shown in Fig. 4B, the intestinal tissues in the X-ray, X-ray + SP, and Curcumin + X-ray groups showed severe destruction of crypt structure. In contrast, the radiation-induced intestinal damage of irradiated mice pretreated with SP@Curcumin was significantly lower (Fig. 4C). In addition, we evaluated the proliferation of intestinal epithelial cells using a Ki67 immunohistochemical staining assay. The number of Ki67-positive cells in the SP@Curcumin + X-ray group was significantly higher than in other groups, indicating that SP@Curcumin treatment could promote cell proliferation in irradiated mice. In addition to permanently damaging the epithelial barrier, enhanced ROS production induced by the high-dose radiation can also induce inflammatory responses, accompanied by the increased expression of proinflammatory cytokines, such as tumor necrosis factor α (TNFα) and interleukin-6 (IL-6) (*40*). Compared with other groups, SP@Curcumin treatment attenuated the expression of both TNFα and IL-6. The expression of TNFα and IL-6 of the curcumin-treated group was reduced, but it was still higher than that of the SP@Curcumin group. Thus, SP@Curcumin demonstrated its capability to protect normal intestinal tissues from radiation-induced damages, thereby highlighting its potential as a robust GI tract radioprotector.

### Chemoradiotherapy of colon cancer

Effective inhibition of tumor progression and reduction of radiation-induced normal tissue damage are of great significance in clinical radiotherapy. In addition to scavenging ROS and exhibiting anti-inflammatory effects, curcumin also has anticancer activity, including the inhibition of the survival, proliferation, and angiogenesis of various tumor cells. Curcumin exerts its effects by regulating the multiple cell signaling pathways, which are crucial in cancer development and progression (*41*, *42*). Therefore, we further explored the possibility of introducing SP@Curcumin in conventional radiotherapy for colon cancer to simultaneously achieve radioprotection to normal tissues and synergistic chemotherapy/radiotherapy effects on tumors. First, the in vitro antitumor capability of SP@Curcumin was evaluated on CT26 colon cells using a calcein acetoxymethyl ester/propidium iodide (calcein-AM/PI) live/dead staining assay (fig. S15). The cells in the control and SP groups showed high viability with strong green fluorescence (fig. S15A). On the contrary, the free curcumin treatment group and the SP@Curcumin treatment group exhibited apparent cell-killing effects, with approximately 46 and 42% cell death (red fluorescence), respectively (fig. S15B). Furthermore, the cell inhibition rate increased to 73% after SP@Curcumin + X-ray treatment, indicating the synergistic tumor-killing effects of SP@Curcumin treatment.

**Fig. 5.**
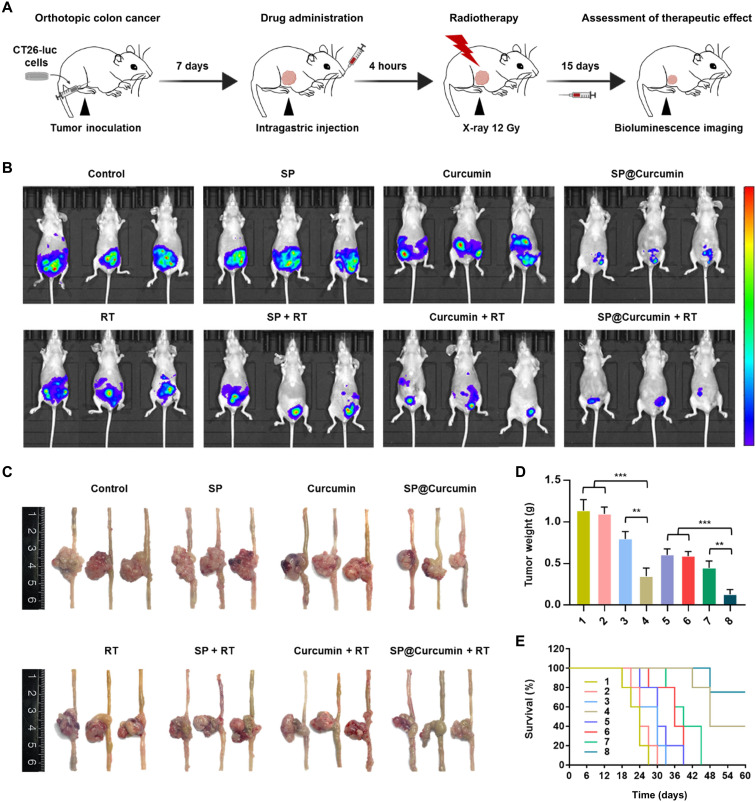
SP@Curcumin-mediated chemoradiotherapy of colon cancer. (**A**) Schematic illustration of combining chemotherapy with radiotherapy to inhibit tumor progression in the orthotopic mouse model of colon cancer. (**B**) Bioluminescence images of mice (*n* = 3) on 15 days after various treatments. (**C**) Photographs of the dissected ceca with tumors of mice on 15 days after different treatments. (**D**) Tumor weights of mice on 15 days in different groups (*n* = 3). (**E**) Survival curves of mice (*n* = 5) with orthotopic colon cancer after various treatments. Treatments: 1, Control; 2, SP; 3, Curcumin; 4, SP@Curcumin; 5, RT; 6, SP + RT; 7, Curcumin + RT; and 8, SP@Curcumin + RT. Photo credit: Danni Zhong, Zhejiang University.

To study the SP@Curcumin-mediated chemoradiotherapy in vivo, we constructed an orthotopic mouse colon tumor model by injecting luciferase-transfected CT26 cells (CT26-luc cells) into the cecum mucosa of nude mice (Fig. 5A). One week after tumor inoculation (day 0), the mice were randomly divided into eight groups: 1, Control; 2, SP; 3, Curcumin; 4, SP@Curcumin; 5, radiotherapy (RT); 6, SP + RT; 7, Curcumin + RT; and 8, SP@Curcumin + RT. At day 15, the luciferase signals of CT26-luc cells were noninvasively monitored by bioluminescence imaging to evaluate the therapeutic effects. The results of bioluminescence imaging showed that, unlike other nonradiotherapy groups, SP@Curcumin alone significantly inhibited tumor growth, and the SP@Curcumin-mediated chemoradiotherapy group had the lowest residual tumor signal (Fig. 5B and fig. S16). These results were also confirmed by the tumor weights measured at the end of these treatments. Mice treated with SP@Curcumin exhibited significantly less tumor growth compared to the control group, while the mice treated with SP@Curcumin + RT showed almost complete tumor eradication, with a tumor growth inhibition rate of 88.7% (Fig. 5, C and D). Meanwhile, as shown in Fig. 5E, CT26 tumor-bearing mice treated with SP@Curcumin survived for more than 60 days after tumor inoculation, with the highest survival rate of 80%, whereas the life span of mice treated with curcumin or radiation alone was only 33 to 45 days.

**Fig. 6.**
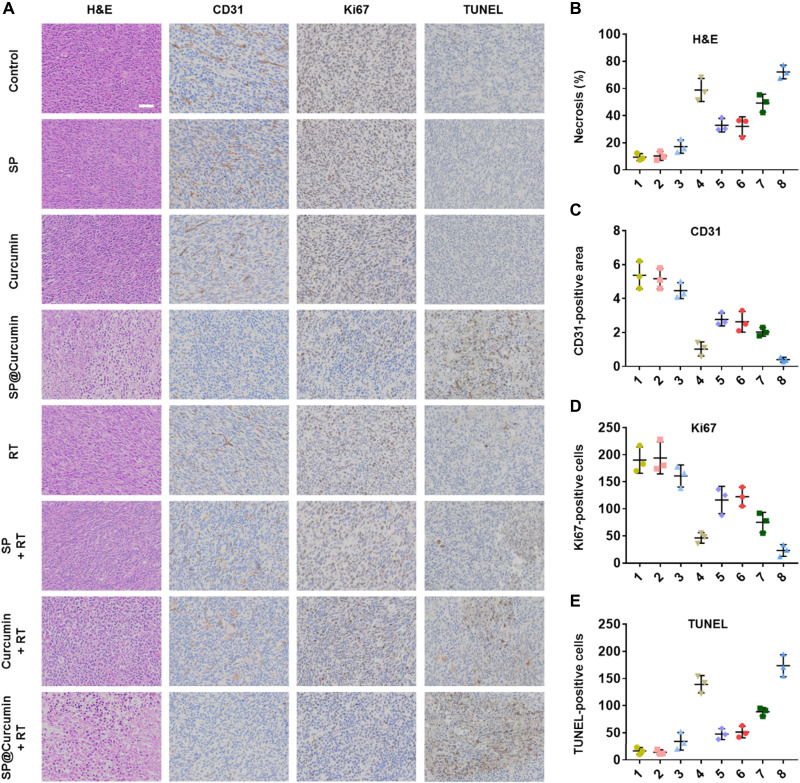
Immunohistochemical evaluation of the effect of synergistic chemoradiotherapy. (**A**) Representative H&E, CD31, Ki67, and TUNEL staining images of tumor tissues. Scale bar, 50 μm. Quantitative analysis of (**B**) necrosis, (**C**) CD31-positive area, (**D**) Ki67-positive cells, and (**E**) TUNEL-positive cells on 15 days after different treatments. Treatments: 1, Control; 2, SP; 3, Curcumin; 4, SP@Curcumin; 5, RT; 6, SP + RT; 7, Curcumin + RT; and 8, SP@Curcumin + RT.

To further evaluate the chemoradiotherapeutic efficacy of SP@Curcumin, hematoxylin and eosin (H&E) staining and immunohistochemical analysis [CD31, Ki67, and terminal deoxynucleotidyl transferase–mediated deoxyuridine triphosphate nick end labeling (TUNEL)] were carried out (Fig. 6A). The H&E staining assay revealed that most tumor cells in the SP@Curcumin + RT group were damaged, with a necrosis rate of 72.1% (Fig. 6B). Specifically, the expression of CD31 and Ki67 decreased after SP@Curcumin + RT treatment (Fig. 6, C and D), while the number of TUNEL-positive cells was the highest in the SP@Curcumin + RT group (Fig. 6E). These results revealed that SP@Curcumin-mediated chemoradiotherapy effectively inhibited tumor growth by suppressing tumor proliferation and vascularization and thus inducing apoptosis.

### Anti-inflammatory effects against intestinal inflammation

**Fig. 7.**
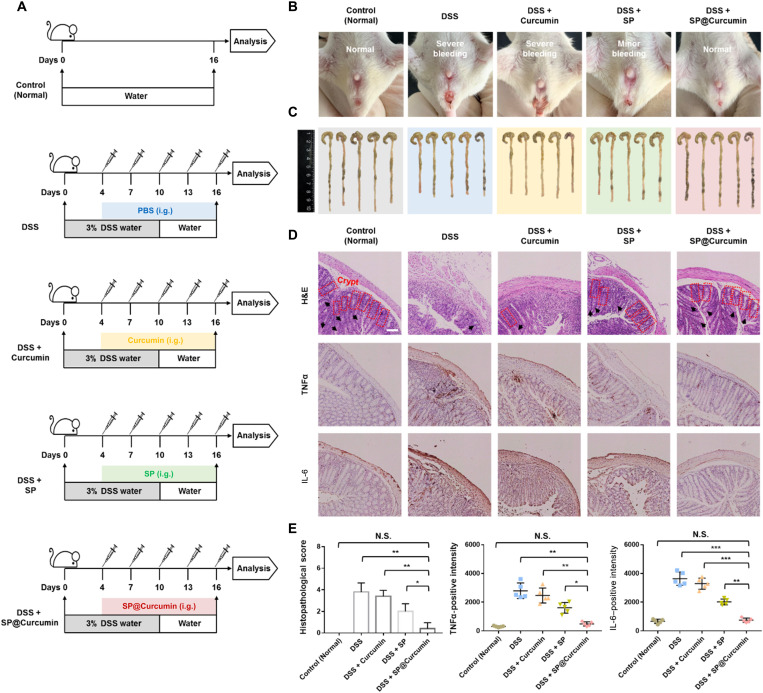
SP@Curcumin-mediated anti-inflammatory effects against intestinal inflammation. (**A**) Schematic illustration of SP-mediated anti-inflammatory effects against DSS-induced colitis in mice. i.g., intragastric. (**B**) Representative photographs of rectal bleeding of mice on 16 days in different groups (*n* = 5). (**C**) Photographs of the dissected colons of mice on 16 days in different groups. (**D**) Representative H&E, TNFα, and IL-6 staining images of colons. Red dotted lines indicate the crypts, and black arrows indicate the goblet cells. Scale bar, 100 μm. (**E**) Histopathological score and TNFα- and IL-6–positive intensity on 16 days in different groups. Data are means ± SD; *n* = 5; Student’s two-tailed *t* test, not significant (N.S.) *P* ≥ 0.05, *P < 0.05, **P < 0.01, and ***P < 0.001. Photo credit: Danni Zhong, Zhejiang University.

As discussed above, SP@Curcumin effectively scavenged ROS, thereby reducing the inflammatory responses induced by the high-dose radiation. We subsequently investigated the anti-inflammatory ability of this microalgal biomass–based drug delivery strategy in the treatment of inflammatory bowel disease (IBD), a chronic and idiopathic inflammatory set of conditions, which includes Crohn’s disease and ulcerative colitis (UC) (*43*). A dextran sulfate sodium (DSS)–induced colitis mouse model was used to evaluate the anti-inflammatory capability of SP@Curcumin. Mice were divided into five groups (*n* = 5): Control, DSS, DSS + Curcumin, DSS + SP, and DSS + SP@Curcumin. Mice in the DSS groups were fed 3% DSS for 10 days to allow the formation of UC. The drug-treated groups were intragastrically administered with free curcumin, SP, or SP@Curcumin five times with the same interval (Fig. 7A). During treatment, the DSS and the DSS + Curcumin groups had obvious body weight loss compared with the control group. However, the SP@Curcumin-treated mice lost less weight than other DSS groups (fig. S17). In addition, severe rectal bleeding was found in the DSS and DSS + Curcumin groups, whereas no abnormalities were observed in the control and SP@Curcumin groups (Fig. 7B). In addition, the length of colon was another biological indicator of intestinal pathology in acute colitis. The average colon lengths in the DSS (7.2 ± 0.1 cm) and DSS + Curcumin (7.3 ± 0.3 cm) groups were remarkably shorter than that of the control group (8.9 ± 0.4 cm), indicating a marked intestinal pathological condition induced by DSS treatment (Fig. 7C and fig. S18). However, the colon length in the DSS + SP@Curcumin group recovered to 8.7 ± 0.4 cm, indicating that SP@Curcumin treatment could relieve the symptoms of DSS-induced colitis.

The colitis symptoms of mice, including weight loss, gross bleeding, and reduced colon length, were somewhat relieved after repeated SP treatment. It has been reported that the microalgal biomass and its extracts, when used as nutritional supplements, can alleviate the IBD symptoms in DSS-induced colitis murine models and human clinical trials (*44*–*46*). Therefore, we hypothesized that the anti-inflammatory effect of SP group might be related to the repeated gavage, similar to the addition of microalgae in the feed. In addition, we examined the ROS levels of colon sections using a 2,7-dichlorofluorescin diacetate (DCFH-DA) staining assay. Strong green fluorescence signals resulting from the ROS expression were observed in the DSS and DSS + Curcumin groups (fig. S19). However, the colon tissues in the SP@Curcumin group showed a much weaker ROS fluorescence signal, indicating that SP@Curcumin had a better ROS scavenging capability than free curcumin in vivo. We next performed the H&E staining assay to evaluate the histopathological changes. H&E-stained sections of colons in the DSS-treated group displayed severe histological damage with irregular epithelial structure, crypt loss, and depleted goblet cells (Fig. 7D). Conversely, mice treated with SP@Curcumin showed normal colonic histology with an intact surface epithelium and crypt structure. These findings also correspond to the histopathological scores, in which the DSS-treated groups showed the highest score and the core significantly decreased after SP@Curcumin intervention (Fig. 7E). Furthermore, the expression of proinflammatory cytokines, including TNFα and IL-6, significantly increased in mice with colitis, whereas the DSS + SP@Curcumin–treated group showed the similar expression to that of nontreated mice (Fig. 7E). These combined results demonstrated that SP@Curcumin treatment could serve as an effective anti-inflammatory strategy with a broad spectrum of anti-inflammatory effects because this treatment could effectively inhibit the inflammatory responses in two mouse models, including radiation-induced intestinal inflammation and DSS-induced colitis.

Last, we evaluated the long-term biosafety of SP@Curcumin after intragastrical administration for up to 30 days. Twenty healthy mice were divided into four groups (*n* = 5): Control, SP, Curcumin, and SP@Curcumin, followed by intragastrical administration with phosphate-buffered saline (PBS), SP, curcumin, or SP@Curcumin, respectively. No significant differences in blood routine and blood biochemistry parameters were observed in the three different types of drug treatment groups compared with the control group (fig. S20). In addition, the H&E-stained histological sections of major organs (heart, liver, spleen, kidney, and lung) from all groups revealed no detectable damage or any inflammatory lesions (fig. S21). Together, the evidence indicates that both SP and SP@Curcumin have good biosafety in vivo.

## DISCUSSION

We used the natural cyanobacterium SP as a drug carrier to efficiently load and deliver curcumin to improve the bioavailability and retention time of the drug in the GI tract, thereby improving the therapeutic effect. In terms of the formulation, we used a one-step strategy to prepare SP@Curcumin with a high drug loading efficiency. Such a straightforward process for the preparation of SP@Curcumin could easily be scaled up in the laboratory settings. In addition, the microalgal carriers effectively protected the loaded drugs from damage by gastric acid, and the unique helical structure of these carriers enabled their preferential accumulation in the intestinal tissues for long-term drug release. Moreover, SP@Curcumin could be tracked noninvasively in vivo through the fluorescent chlorophyll in microalgal carriers. On the one hand, our results indicated that SP@Curcumin exerted radioprotective effects on normal intestinal tissues and combined chemoradiotherapeutic effects on colon tumors. On the other hand, SP@Curcumin exhibited a strong anti-inflammatory effect against DSS-induced colitis, demonstrating its excellent therapeutic performance on intestinal inflammatory diseases. Both in vitro and in vivo studies showed no systemic side effects after the treatment of SP@Curcumin. Therefore, the treatment by microalgal biomass–based multifunctional platform reported here can serve as a promising theranostic strategy for the management of colon diseases.

The current stage of biomedical applications of microalgal carrier–based drug delivery is still in its infancy, especially in the treatment of GI diseases. To date, microalgal biomass–based drug delivery systems have mostly been studied in vitro, with only a few in vivo studies reported recently (*31*–*33*, *47*). In the early stage, Yasa *et al.* (*29*) attached drug-loaded magnetic nanoparticles to the surface of microalgae through noncovalent electrostatic interactions, thus realizing efficient drug loading and magnetically targeted drug delivery. Despite these achievements, the key challenges such as surface interactions, nanomaterial degradation, and immunotoxicity limited the microalgae-based platform to be used only in in vitro studies. A recent study reported by Qiao *et al.* (*47*) addressed these problems by modifying microalgae with highly biocompatible cell membranes to treat tumors by intravenous administration. Nevertheless, the safety and reliability of intravenous administration of microalgae for clinical use remain difficult and controversial. Although the study reported by Yan *et al.* (*32*) promoted the possibility of clinical applications of the microalgal carriers by oral delivery of magnetic microalgal swimmers for magnetic resonance imaging/fluorescence tracking inside of rodent stomachs, the biomedical applications of microalgae-based delivery systems and their therapeutic capabilities in intestinal disease models have yet to be examined.

In this work, we used a microalgal biomass–based oral drug delivery strategy for intestinal disease treatment. We set out to address the concerns regarding the feasibility and biosafety of microalgal agents for medical purposes. From one perspective, we have effectively loaded small-molecule drugs in microalgae by a one-step approach, integrating the excellent functions of both elements to achieve multifunctionality. The designed microalgae-based drug delivery system has been directly used for oral administration to treat intestinal diseases, without the need for additional chemical modifications to achieve in vivo targeting, as required in intravenous administration. As a result, the abovementioned shortcomings associated with the intravenous administration of other functional materials and microalgae carriers themselves could thus be avoided. In particular, we have highlighted several important advantages of the microalgal delivery system as oral therapeutics, including (i) the protection of loaded drugs from degradation in harsh conditions, (ii) the long-lasting intestinal retention effects, (iii) the noninvasive in vivo imaging capability based on the natural fluorophores, and (iv) the excellent biodegradability. From another perspective, we have demonstrated the accessibility and versatility of the microalgae-based drug delivery system in mouse models for the treatment of a variety of intestinal diseases, including the protection of the intestinal tract under radiation exposure, synergistic chemoradiotherapy for colon cancer, and inflammation resolution in acute colitis. Nevertheless, to realize the clinical translation of these microalgal agents in the future, several important aspects still need to be explored in future studies: (i) selection of the suitable microalgal carriers for the corresponding disease models, (ii) optimization of the drug loading process of multiple drug molecules, (iii) multimodal therapeutic effects, and (iv) systematic safety assessment in large animal models, especially in nonhuman primate models. As a renewable resource with intriguing properties, microalgal biomass may open up new avenues for more innovative diagnostic and treatment options.

## MATERIALS AND METHODS

### Characterization and synthesis of SP@Curcumin

SP and Zarrouk medium were purchased from Guangyu Biological Technology (Shanghai, China). SP was cultured in Zarrouk medium at 30°C in a lighted environment. SP samples were collected by centrifugation at 3260*g* for 10 min and washed three times with DI water. Curcumin powder (3AChemicals, Shanghai, China) was dissolved in ethanol at a high concentration for preservation and then diluted with DI water to a series of concentrations before use. For the synthesis of SP@Curcumin, 1 ml of curcumin solutions (25, 50, 100, 200, 400, 800, and 1600 μg/ml) was added to 10 ml of SP (50 μg/ml) suspensions. The mixtures were stirred for 12 hours. Last, the as-prepared SP@Curcumin was collected by centrifugation at 3260*g* for 10 min and then stored in DI water for further experiments. For lyophilization, the obtained SP@Curcumin samples were suspended in DI water, frozen (−20°C for 24 hours), and then freeze-dried for 24 hours (collector temperature, −80°C; vacuum, 5 Pa) in the Labconco Mobile Freeze Dryer (710611040, Kansas City, MO, USA). The amount of loaded curcumin was quantified by the absorbance of the collected supernatants at 480 nm by UV-vis spectra, in combination with a standard curve of curcumin solutions. Drug loading efficiency (DLE%) was defined as (the amount of loaded curcumin/the amount of curcumin in the initial curcumin loading solution) × 100%. Bright-field and fluorescence microscope images were captured with an optical microscope (Zeiss, Oberkochen, Germany) connected to a PC with a charge-coupled device camera. SEM images of SP were acquired using an SEM (SU-8010, Hitachi, Japan). The samples were first fixed in 2.5% glutaraldehyde in phosphate buffer (0.1 M, pH 7.0) for more than 4 hours and then washed with phosphate buffer (0.1 M, pH 7.0) for three times. Then, the samples were fixed in 1% OsO_4_ in phosphate buffer for 1 to 2 hours and washed three times with phosphate buffer. For dehydration, the samples were first dehydrated sequentially with different concentrations of ethanol (30, 50, 70, 80, 90, and 95%) and finally dehydrated with a Hitachi HCP-2 critical point dryer. The dehydrated samples were coated with gold-palladium in a Hitachi model E-1010 ion sputter for 4 to 5 min and observed in a Hitachi SU-8010 SEM. UV-vis spectra were recorded with a UV-2600 spectrophotometer (Shimadzu, Kyoto, Japan).

### Drug release study

SP@Curcumin samples were suspended in 2 ml of PBS at different pH values (1.8 and 6.5) and incubated at 37°C. At the given time points, the supernatants were collected using centrifugation at 3260*g* for 10 min and then replaced by an equal volume of fresh PBS. The cumulative amounts of curcumin release at different time points were measured using UV-vis spectra.

### MTT assay

Normal rat small intestinal epithelial cells [IEC-6 cells; American Type Culture Collection (ATCC), no. CRL-1592, passage numbers: 10 to 15] were cultured in DMEM supplemented with 10% fetal bovine serum, 1% antibiotics [penicillin (100 U/ml) and streptomycin (100 μg/ml)], and bovine insulin (0.1 U/ml) at 37°C under 5% CO_2_ atmosphere. IEC-6 cells were seeded and incubated in 96-well plates (1 × 10^4^ per well) overnight and then incubated with SP/curcumin/SP@Curcumin at predetermined concentrations for 24 hours. Cell viability was measured using a standard MTT assay kit (YEASEN, Shanghai, China).

### DCFH-DA staining assay

IEC-6 cells were seeded in 96-well plates at a density of 1 × 10^4^ cells per well and treated with 100 μl of SP/curcumin/SP@Curcumin (SP, 25 μg/ml; curcumin, 17.4 μg/ml) for 2 hours and then irradiated with x-ray at a dose of 12 Gy. To simulate the retention of SP in the intestine, the cells were first coincubated with 100 μl of SP/curcumin/SP@Curcumin (SP, 25 μg/ml; curcumin, 17.4 μg/ml) for 2 hours, and then the medium was replaced with fresh DMEM. The cells were further incubated in DMEM for 2 hours and x-ray–irradiated (12 Gy). X-ray–treated cells were stained with a DCFH-DA assay kit (YEASEN, Shanghai, China) and imaged under a fluorescence microscope (Zeiss, Oberkochen, Germany) to evaluate the intracellular production of ROS.

### DNA double-strand breaks

IEC-6 cells (1 × 10^5^) were seeded in a 20-mm confocal dish and treated with SP/curcumin/SP@Curcumin (SP, 25 μg/ml; curcumin, 17.4 μg/ml) for 4 hours and then irradiated with x-ray at a dose of 12 Gy. After different treatments, cells were fixed with 4% paraformaldehyde for 30 min, immersed in Triton X-100 for 10 min to rupture the cell membrane, and then blocked with 5% bovine serum albumin for 1 hour at room temperature. Afterward, the cells were incubated with γ-H2AX antibody at 4°C overnight in a dark environment and then incubated in sheep anti-mouse secondary antibody for 2 hours. Last, the cells were imaged under a confocal microscope (Nikon A1, Japan) to evaluate the formation of DNA double-strand breaks. Nuclei and DNA fragmentation were stained by 4′,6-diamidino-2-phenylindole (DAPI) (blue) and γ-H2AX (red), respectively.

### Colony formation assay

IEC-6 cells (1 × 10^5^) were seeded in a six-well plate at a density of 2 × 10^5^ cells per well and treated with SP/curcumin/SP@Curcumin (SP, 25 μg/ml; curcumin, 17.4 μg/ml) for 4 hours, followed by exposure to x-ray at a dose of 12 Gy. Cells (1 × 10^3^) from each treatment group were seeded into six-well plates and cultured for 7 days. Colony formation was analyzed by counting (≥50 cells) after fixing with methanol and staining with Giemsa solution.

### Live/dead cell staining assay

Mouse colon cancer cells (CT26 cells; ATCC, no. CRL-2638, passage numbers: 5 to 9) were seeded in 96-well plates at a density of 1 × 10^4^ cells per well and treated with SP/curcumin/SP@Curcumin (SP, 25 μg/ml; curcumin, 17.4 μg/ml) for 4 hours, followed by exposure to x-ray at a dose of 12 Gy. Live/dead cells were stained using a calcein-AM/PI double stain kit (YEASEN, Shanghai, China) and imaged with a fluorescence microscope.

### Animal experiments

All animal experiments were approved by the Institutional Animal Care and Use Committee of Zhejiang University (2020-844) and were performed under protocols approved by this committee. Female Balb/c mice (6 weeks of age) were purchased from Shanghai Slac Laboratory Animal Co. Ltd.

### Fluorescence imaging and biodistribution

Female Balb/c nude mice (6 weeks, *n* = 3) were administered intragastrically with 250 μl of SP@Curcumin (SP, 1.7 mg/ml; curcumin, 1.2 mg/ml) and imaged with IVIS Lumina LT Series III (PerkinElmer, USA) at different time points before and after administration (0, 0.5, 1, 2, 4, 6, 8, and 24 hours). The test parameters were consistent with the fluorescence channel of chlorophyll, with an excitation wavelength of 605 nm and an emission wavelength of 615 to 665 nm (Cy5.5). At predetermined time points, mice (*n* = 3) were sacrificed, and all major organs and tissues, including heart, liver, spleen, lung, kidney, bladder, and GI tract of mice, were carefully collected and photographed with IVIS Lumina LT Series III. After intragastric administration, the different parts of the GI tract (stomach, duodenum, jejunum, ileum, cecum, and colon) of the mice were removed. The morphological changes of the SP in vivo were imaged using optical microscopy and SEM.

### Pharmacokinetic study

Pharmacokinetic profiles of oral administration of free curcumin and SP@Curcumin were determined by high-performance liquid chromatography (HPLC). Female Balb/c nude mice (6 weeks) were injected intragastrically with 250 μl of curcumin (1.2 mg/ml) and SP@Curcumin (SP, 1.7 mg/ml; curcumin, 1.2 mg/ml). The blood samples (*n* = 3) collected at different time points (5 min, 15 min, 30 min, 1 hour, 2 hours, 3 hours, 4 hours, and 6 hours after administration) were centrifuged at 2000*g* for 10 min, and the plasma was removed and stored at −20°C. Then, 400 μl of methanol was added to 100 μl of plasma to precipitate proteins. The mixture was placed at −20°C for 4 hours, vortex-mixed for 2 min, and centrifuged at 15,000*g* for 15 min. Last, the supernatant was analyzed with a Shimadzu LC-20A HPLC system (Shimadzu, Kyoto, Japan). The HPLC analysis was performed on an AQ-C18 analytical column (250 mm by 4.6 mm, 5 μm), with methanol–0.5% formic acid (75:25, v/v) as the mobile phase. The flow rate was set at 1.0 ml/min, and the detection wavelength was set at 425 nm.

### Radioprotection of intestinal tissue

Female Balb/c mice (6 weeks) were randomly divided into four groups (*n* = 5)—Control, SP, Curcumin, and SP@Curcumin—and were injected intragastrically with 250 μl of PBS, SP (1.7 mg/ml), curcumin (1.2 mg/ml), and SP@Curcumin (SP, 1.7 mg/ml; curcumin, 1.2 mg/ml), respectively. Four hours after the intragastrical administration, mice were exposed to x-ray at a dose of 12 Gy. Seven days after the treatments, mice were euthanized and the ileum tissues in different groups were excised, fixed in PBS with 5% formaldehyde, and then sectioned. Ileum sections were then stained for H&E, Ki67, TNFα, and IL-6. All sections were examined with a virtual slide microscope (Olympus VS120, Olympus Life Sciences, Waltham, MA, USA).

### Chemoradiotherapy of colon cancer

To obtain CT26-luc cells, exponentially growing CT26 cells at 30% confluency were infected with lentivirus (Ubi-MCS-firefly_Luciferase-IRES-Puromycin, GeneChem, Shanghai, China) according to the product guideline. CT26-luc cells were collected and resuspended in PBS. Female Balb/c nude mice (6 weeks) were anesthetized with isoflurane [2% (v/v) isoflurane in oxygen-enriched air]. A small left abdominal flank incision was made, and 1 × 10^6^ CT26-luc cells in 25 μl of PBS were injected into the cecum mucosa of mice for preparation of a mouse model of orthotopic colon cancer. One week after the tumor inoculation (day 0), mice were randomly divided into eight groups (*n* = 3): 1, Control; 2, SP; 3, Curcumin; 4, SP@Curcumin; 5, RT; 6, SP + RT; 7, Curcumin + RT; and 8, SP@Curcumin + RT. Mice in groups 1 and 5 were injected intragastrically with 250 μl of PBS. Mice in groups 2 and 6 were injected intragastrically with 250 μl of SP (1.7 mg/ml). Mice in groups 3 and 7 were injected intragastrically with 250 μl of curcumin (1.2 mg/ml). Mice in groups 4 and 8 were injected intragastrically with 250 μl of SP@Curcumin (SP, 1.7 mg/ml; curcumin, 1.2 mg/ml). Four hours after the intragastrical administration, mice were exposed to x-ray at a dose of 12 Gy. Mice were intragastrically administered with different drugs on 3, 6, 9, 12, and 15 days. On day 15, mice were intraperitoneally injected with d-luciferin (150 mg/kg), and 10 min later, bioluminescent imaging (BLI) signals of colon tumors were detected by IVIS Lumina LT Series III to assess the therapeutic effects. The BLI intensities of tumors were quantified using Living Image 4.5 software. The mice were then sacrificed, and the ceca with tumors from each group were dissected and photographed with a digital camera (SONY, Japan). The tumors were further sectioned and stained with H&E, CD31, Ki67, and TUNEL.

### Anti-inflammatory effects against intestinal inflammation

Female Balb/c mice (6 weeks) were randomly divided into five groups (*n* = 5): 1, Control (Normal); 2, DSS; 3, DSS + Curcumin; 4, DSS + SP; and 5, DSS + SP@Curcumin. Mice in groups 2 to 5 were fed with 3% DSS aqueous solution instead of drinking water for 10 days for the preparation of DSS-induced colitis mouse model, followed by intragastrical administration of different drugs on days 4, 7, 10, 13, and 16. Mice in group 2 were injected intragastrically with 250 μl of PBS. Mice in group 3 were injected intragastrically with 250 μl of curcumin (1.2 mg/ml). Mice in group 4 were injected intragastrically with 250 μl of SP (1.7 mg/ml). Mice in group 5 were injected intragastrically with 250 μl of SP@Curcumin (SP, 1.7 mg/ml; curcumin, 1.2 mg/ml). To evaluate the anti-inflammatory effect, mice from different groups were sacrificed and the colons were dissected for length measurement as well as for H&E, TNFα, and IL-6 staining.

### Statistical analysis

All statistical analyses were performed using the SPSS software package (SPSS Inc., USA). All error bars used in this study are means ± SD of at least three independent experiments. Statistically significant *P* values are indicated in the figures and/or legends as *P < 0.05, **P < 0.01, and ***P < 0.001.

## Notes

View/request a protocol for this paper from *Bio-protocol*.
