# Supplementary material for: Orally deliverable strategy based on microalgal biomass for intestinal disease treatment
Source: Sci Adv. 2021 Nov 24;7(48):eabi9265. doi: 10.1126/sciadv.abi9265 (PMC8612690; doi:10.1126/sciadv.abi9265)
Supplement: Supplementary file 1 — Figs. S1 to S21 [file sciadv.abi9265_sm.pdf]

Science Advances
AAAS

Supplementary Materials for

Orally deliverable strategy based on microalgal biomass for intestinal disease treatment

Danni Zhong, Dongxiao Zhang, Wei Chen, Jian He, Chaojie Ren, Xingcai Zhang, Na Kong, Wei Tao*, Min Zhou*

*Corresponding author. Email: zhoum@zju.edu.cn (M.Z.); wtao@bwh.harvard.edu (W.T.)

Published 24 November 2021, *Sci. Adv.* **7**, eabi9265 (2021)
DOI: 10.1126/sciadv.abi9265

The PDF file includes:

Figs. S1 to S21

Other Supplementary Material for this manuscript includes the following:

Movie S1

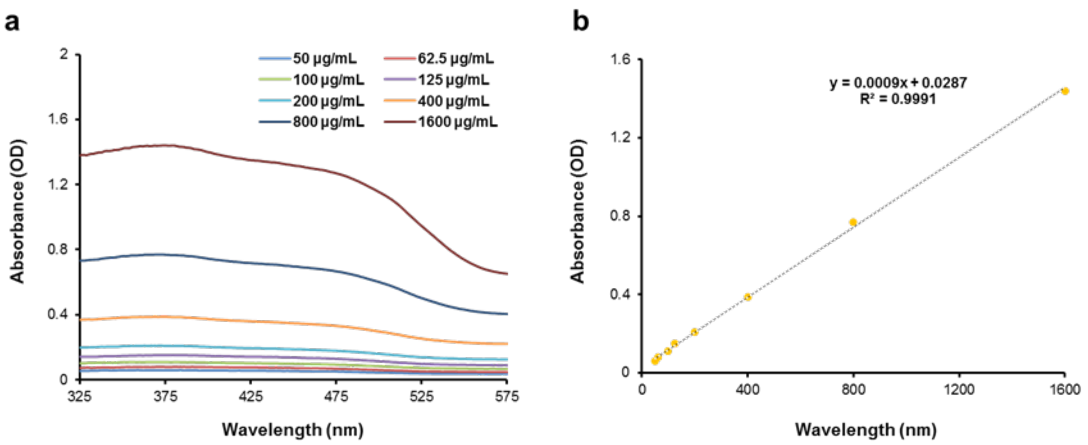

Fig. S1. Standard curve of curcumin solutions calculated using the absorbance at 480 nm. (**a**) UV-Vis spectra of various concentrations of curcumin solutions. (**b**) Standard curve of curcumin solutions calculated using the absorbance at 480 nm.

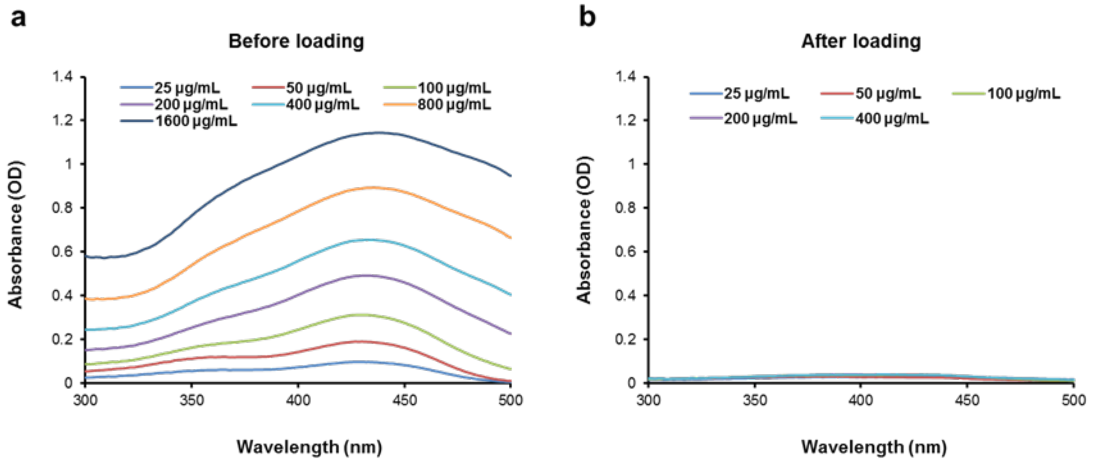

Fig. S2. UV-Vis spectra of the drug solutions before and after incubation with SP. UV-Vis spectra of (**a**) the curcumin solutions with a series of concentrations before loading and (**b**) the culture supernatants after 12 h incubation of curcumin with SP.

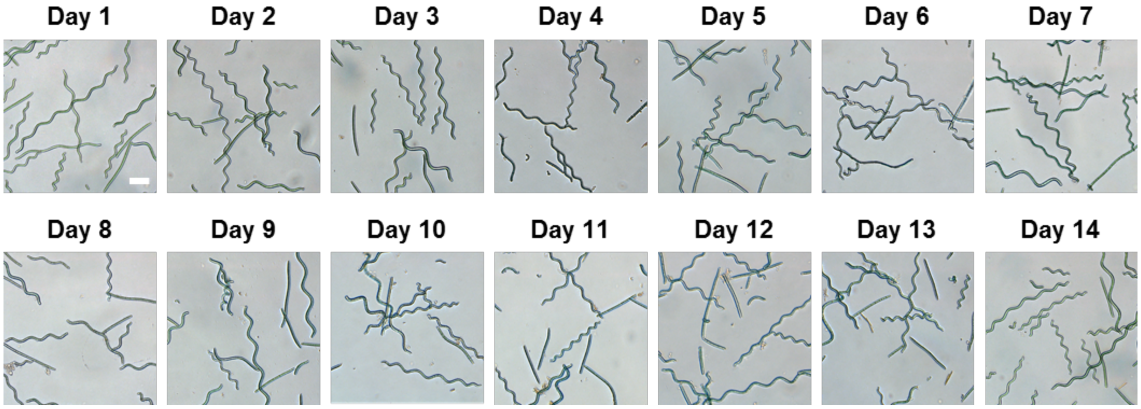

Fig. S3. Bright-field images of SP@Curcumin during two weeks of storage in DI water at 25 °C. Scale bar = 50 μm.

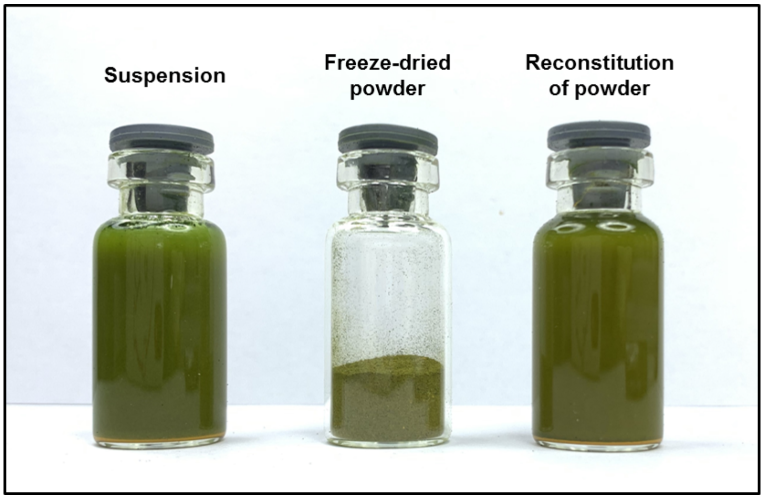

Fig. S4. Different formulations of SP@Curcumin, including suspension, freeze-dried powder, and reconstitution of powder. Photo Credit: Danni Zhong, Zhejiang University.

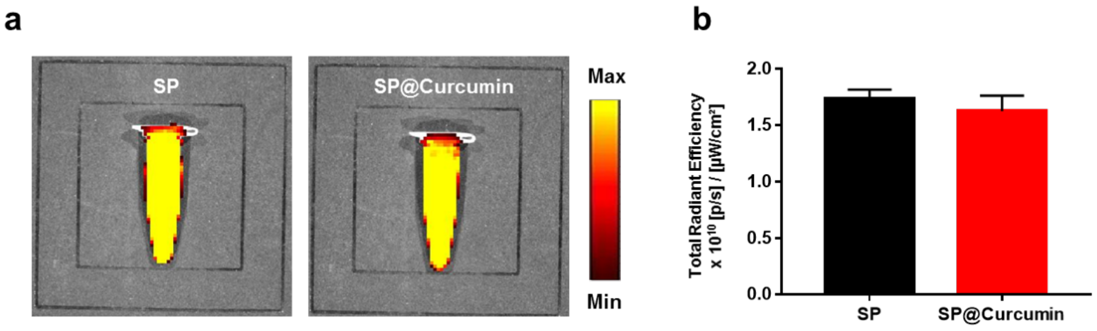

Fig. S5. Fluorescence performance of SP before and after drug loading. (**a**) In vitro fluorescence images of SP and SP@Curcumin under the selected channel (Ex: 605 nm, Em: 615-665 nm, Cy5.5). (**b**) Quantitative analysis of the total fluorescence intensity of SP and SP@Curcumin samples.

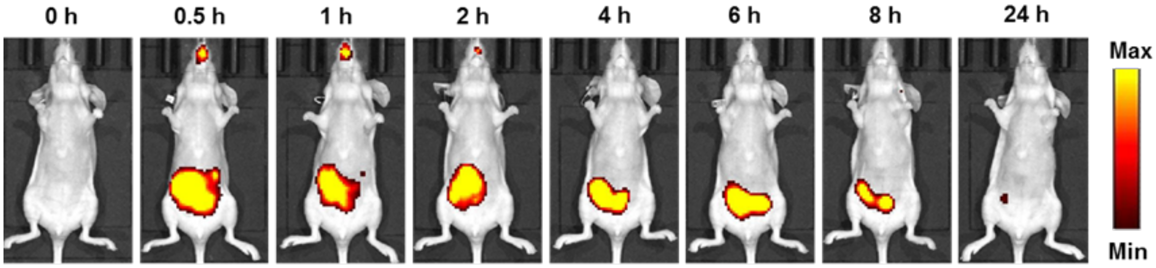

Fig. S6. Time-dependent in vivo fluorescence images of Balb/c nude mice post i.g. administration with SP@Curcumin.

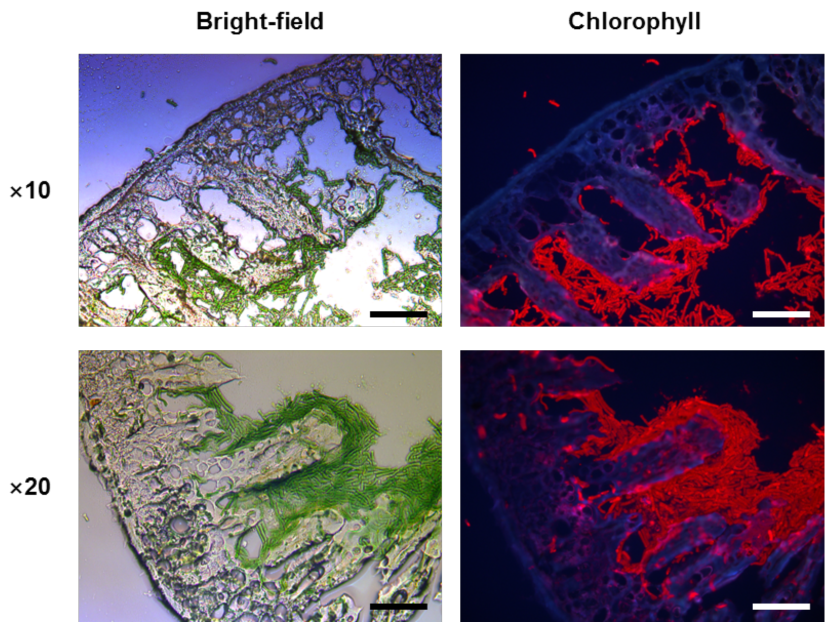

Fig. S7. Bright-field and fluorescence microscope images (red, chlorophyll) of *S. platensis* in the small intestines. The scale bars are 200 and 100 μm for magnification ×10 and magnification ×20, respectively.

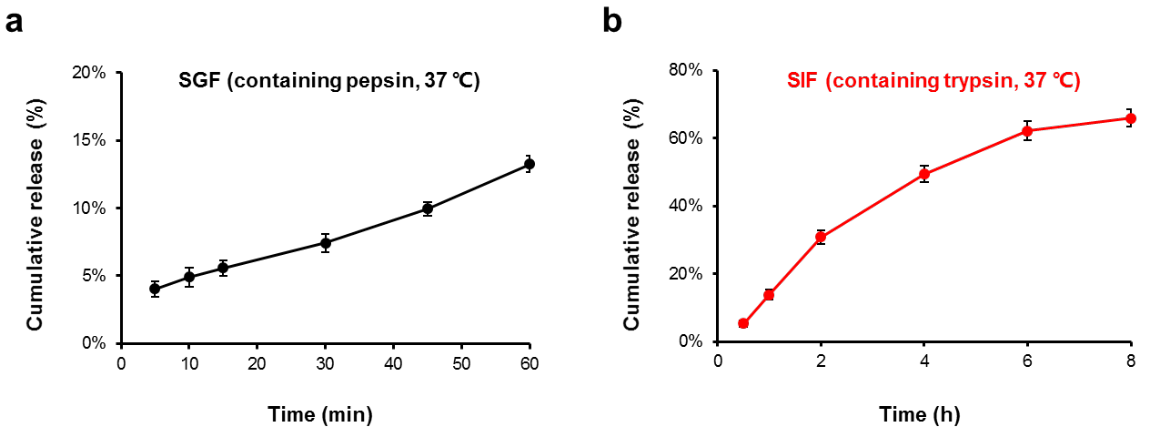

Fig. S8. Cumulative release of curcumin from SP@Curcumin over time in (**a**) SGF containing pepsin (pH 2), and (**b**) SIF containing trypsin (pH 6.8) at 37 °C.

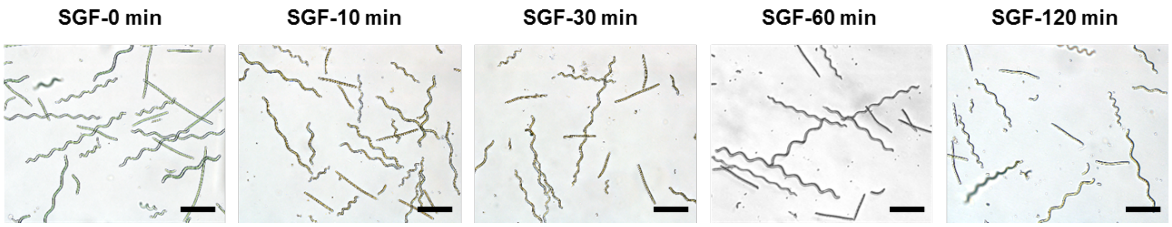

Fig. S9. Bright-field images of SP treated with SGF for 0, 10, 30, 60 and 120 min. Scale bars = 100 μm.

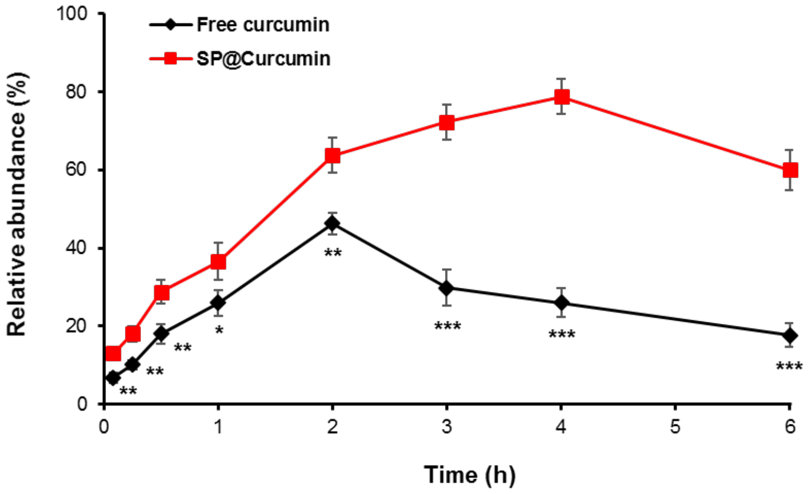

Fig. S10. Time-dependent relative abundance of curcumin in plasma after i.g. administration with free curcumin and SP@Curcumin. Data are means ± SD, n = 3, two-tailed Student's t-test, *P < 0.05; **P < 0.01; ***P < 0.001.

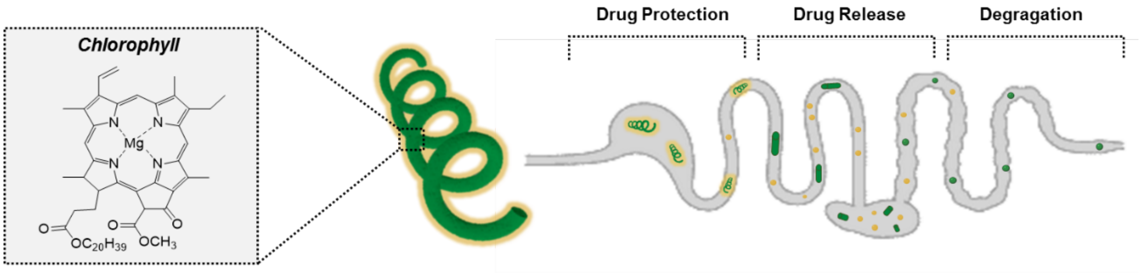

Fig. S11. Schematic illustration of the chlorophyll-based fluorescence imaging and how SP protects the drug from destruction by gastric acid, gradually degrades and releases the drug in the intestinal tract.

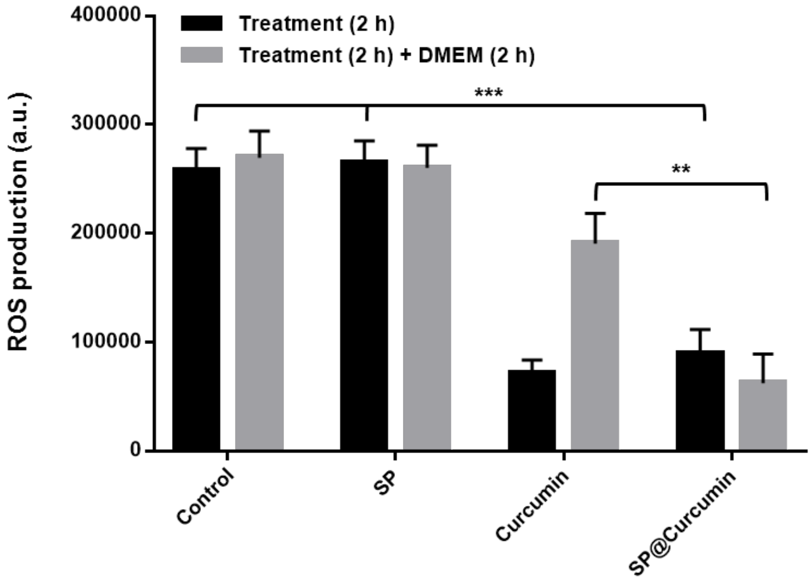

Fig. S12. Quantitative analysis of the fluorescence intensity of the generated ROS after different treatments. Data are means ± SD, n = 3, two-tailed Student's t-test, **$p < 0.01$, ***$p < 0.001$.

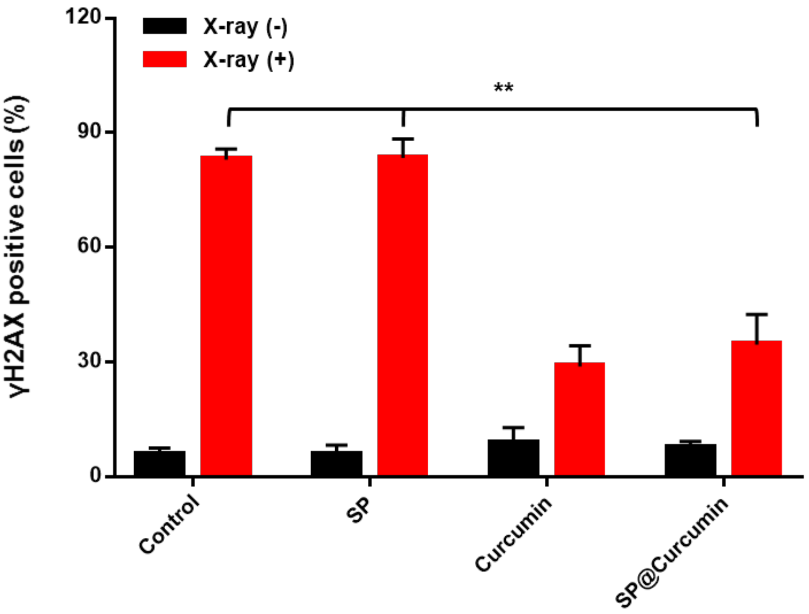

Fig. S13. Quantitative analysis of the percentage of γH2AX-positive cells after different treatments. Data are means ± SD, n = 3, two-tailed Student's t-test, $^{**}p < 0.01$.

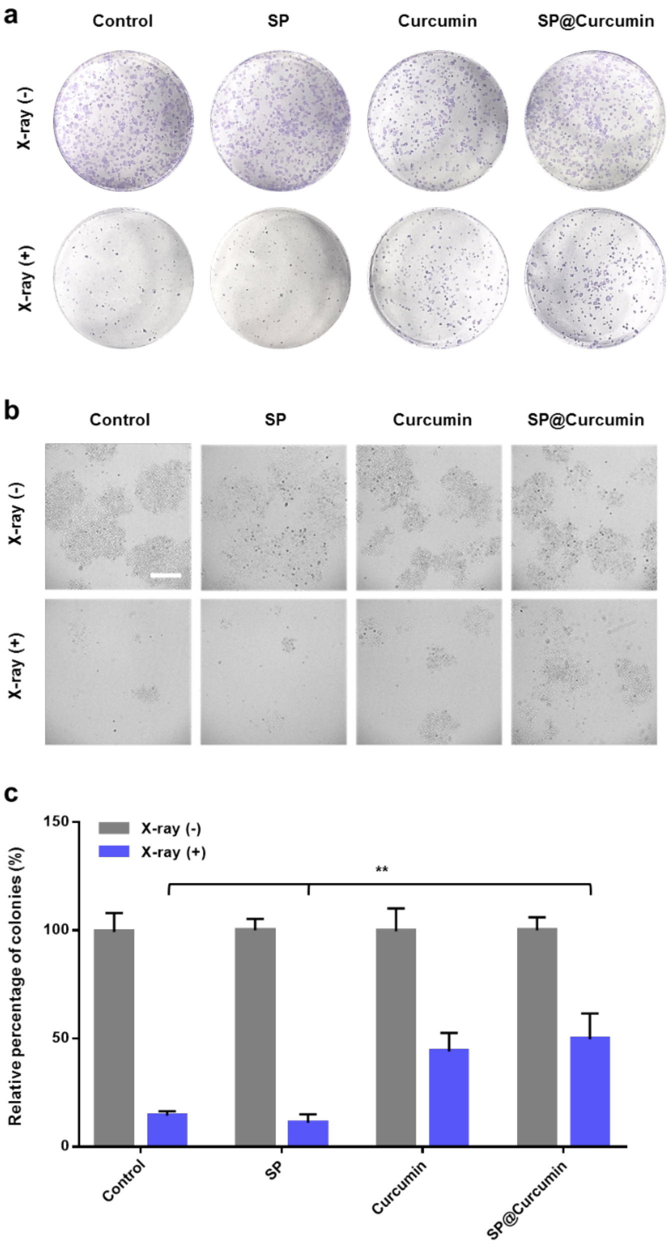

Fig. S14. Colon formation assay of IEC-6 cells after different treatments. (**a**) Representative photographs of colony formation and (**b**) corresponding IEC-6 cell colonies after different treatments. Scale bar = 500 μm. (**c**) Quantitative analysis of the percentage of colony formation after different treatments. Data are means ± SD, n = 3, two-tailed Student's t-test, **p < 0.01.

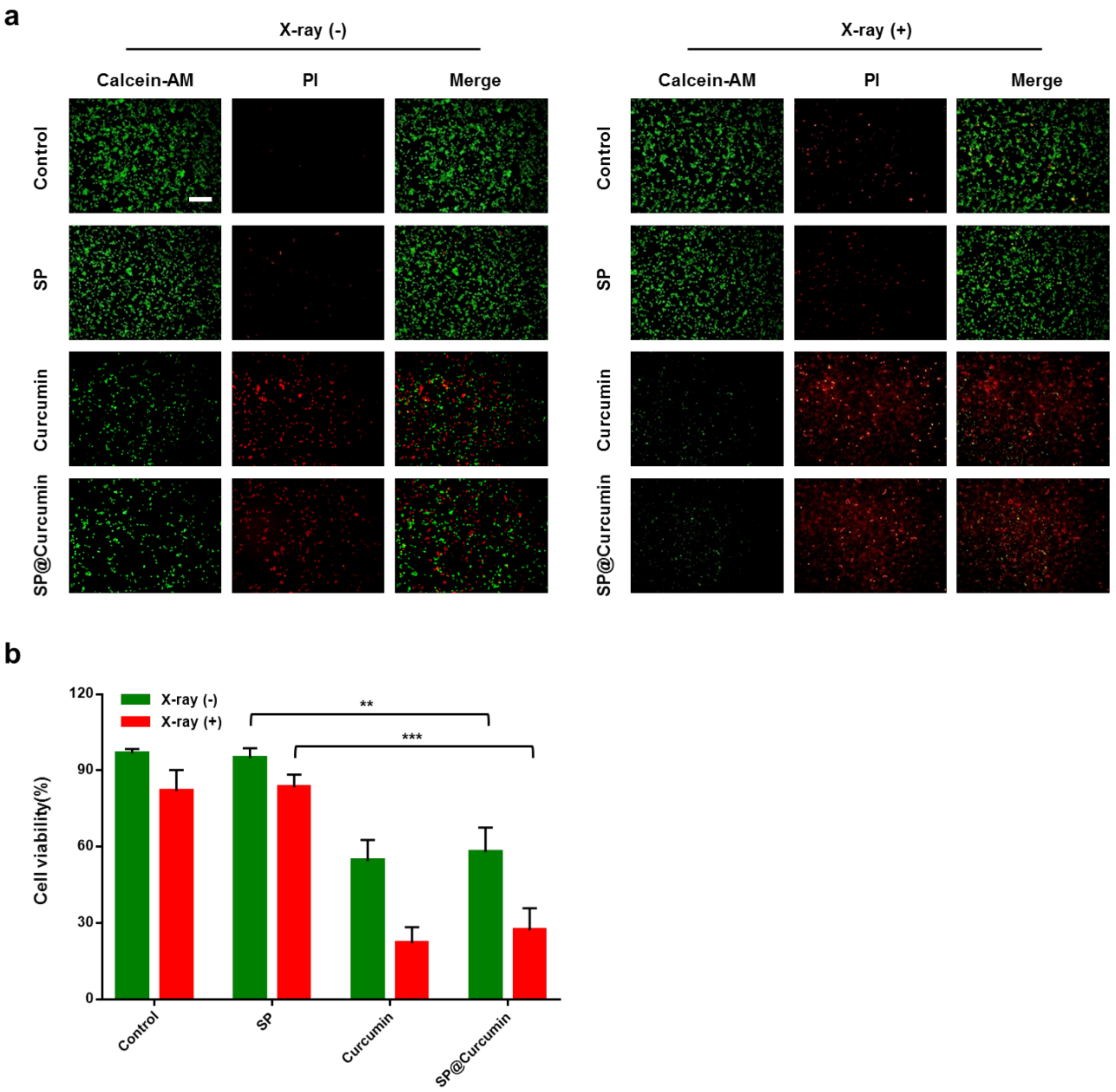

Fig. S15. Anti-cancer effects of SP@Curcumin on CT26 tumor cells. (**a**) Representative images of CT26-luc cells stained with Calcein-AM (green, live cells) and PI (red, dead cells) after different treatments. Scale bar = 200 μm. (**b**) Quantitative analysis of cell viability in the live/dead staining assay after different treatments. Data are means ± SD, n = 3, two-tailed Student's t-test, $^{**}p < 0.01$; $^{***}p < 0.001$.

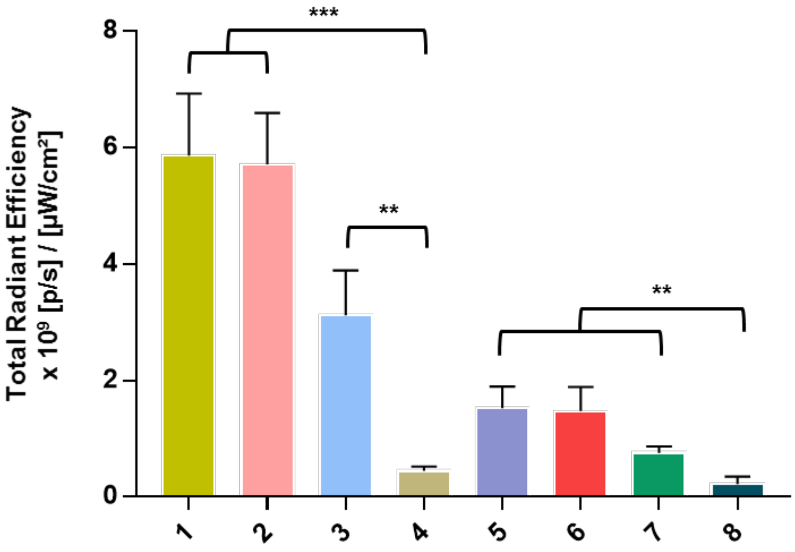

Fig. S16. Quantitative analysis of bioluminescence intensity of mice on 15 d in different groups. Data are means ± SD, n = 3, two-tailed Student's t-test, $^{**}p < 0.01$; $^{***}p < 0.001$.

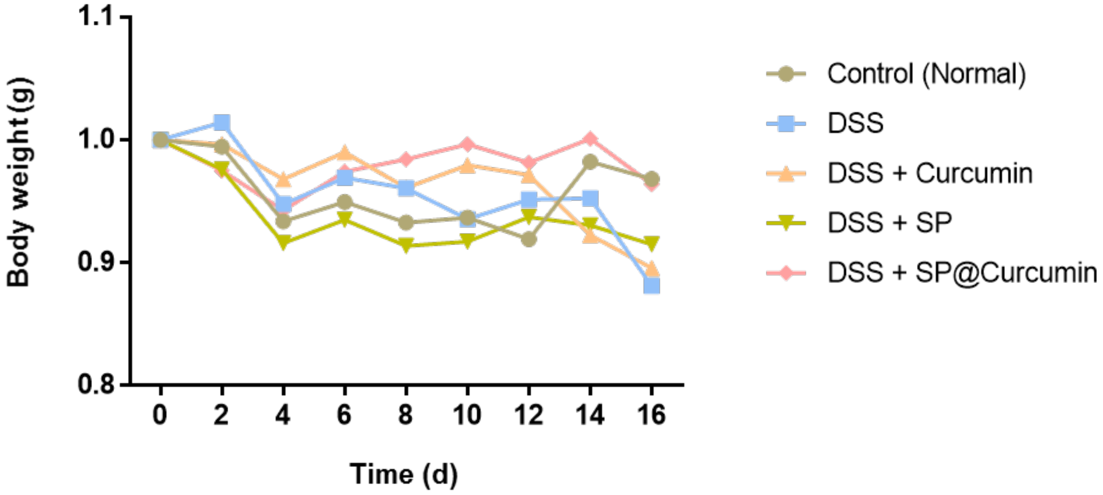

Fig. S17. Body weight loss in different groups in the mouse model of DSS-induced acute colitis.

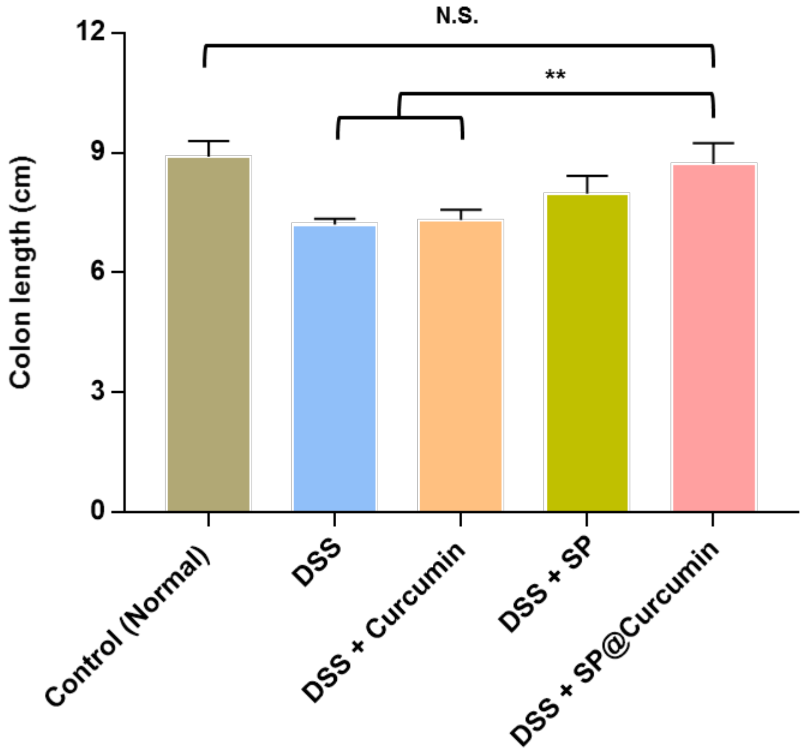

Fig. S18. Quantitative analysis of colon length in the mouse model of DSS-induced acute colitis on 16 d. Data are means ± SD, n = 5, two-tailed Student's t-test, not significant (N.S.) P ⩾ 0.05; **P < 0.01.

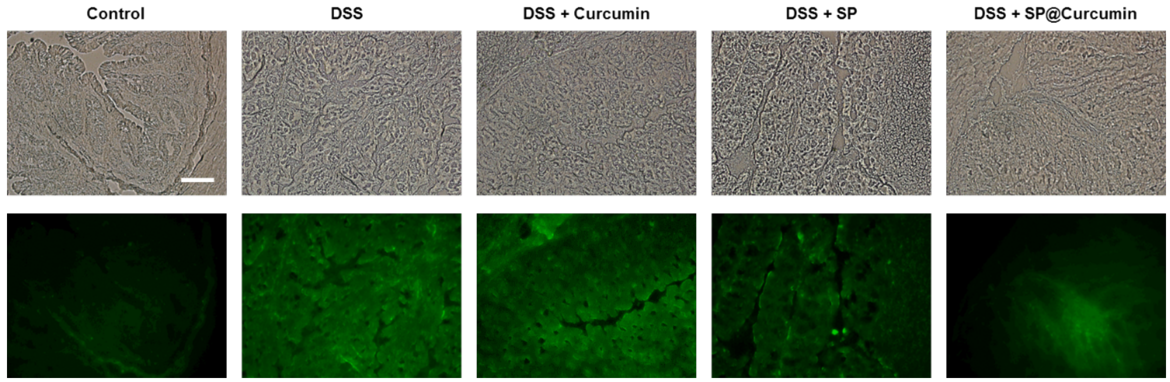

Fig. S19. Fluorescence microscope images of ROS expression of colon tissues after different treatments. Scale bar = 200 μm.

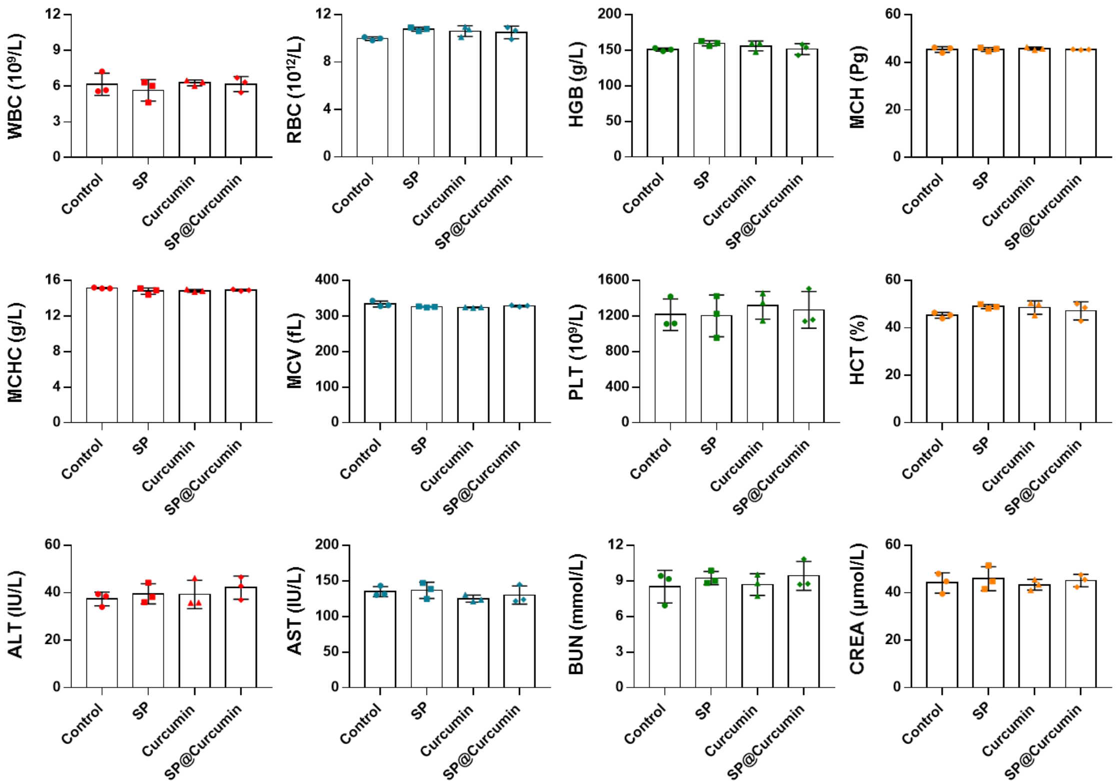

Fig. S20. Blood routine and blood biochemistry tests of the mice 30 days after the i.g. administration with PBS, SP, Curcumin, or SP@Curcumin (n = 3). WBC, white blood cells; RBC, red blood cells; HGB, hemoglobin; MCH, mean corpuscular hemoglobin; MCHC, mean corpuscular hemoglobin concentration; MCV, mean cell volume; PLT, blood platelet; HCT, hematocrit; ALT, alanine transferase; AST, aspartate transferase; BUN, blood urea nitrogen; CREA, creatinine.

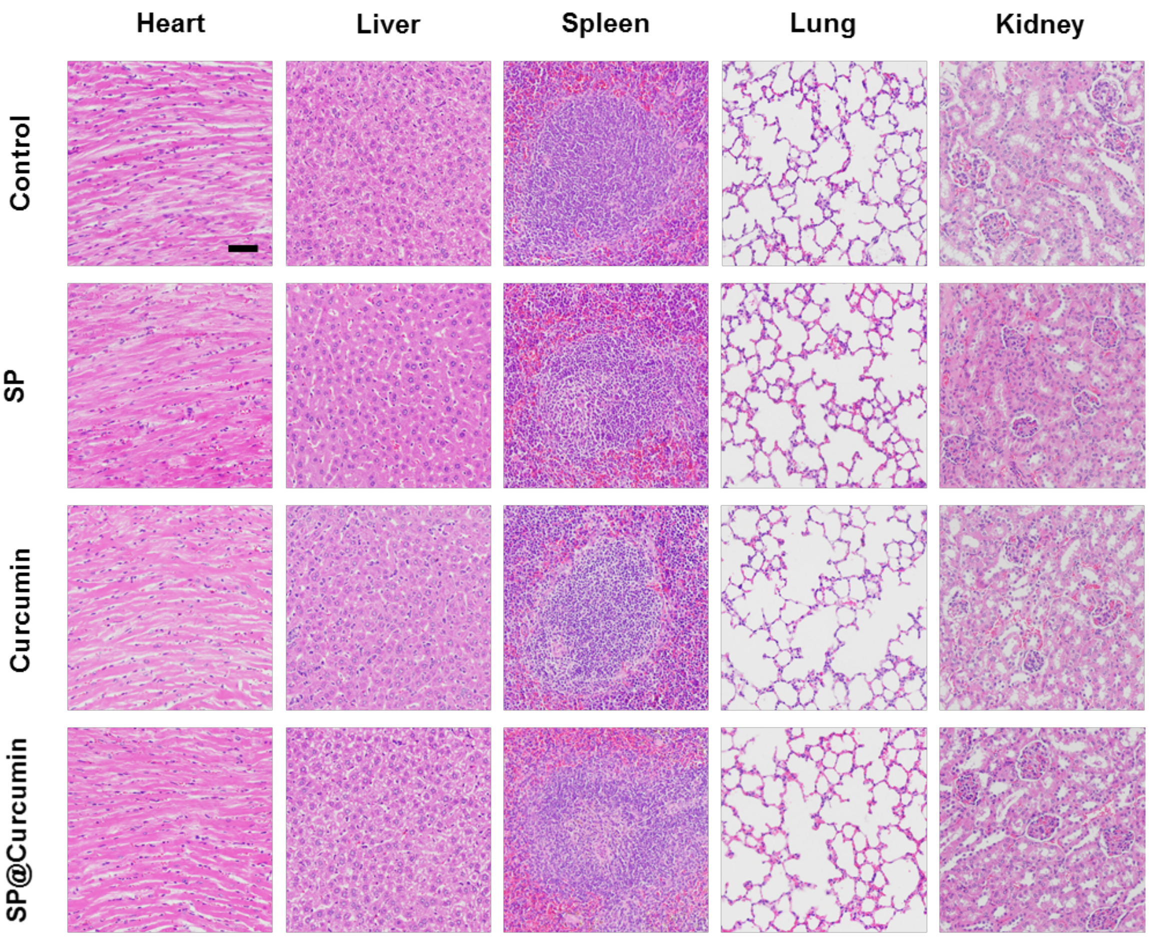

Fig. S21. H&E staining of the major organs (heart, liver, spleen, lung, and kidney) of mice 30 days after the i.g. administration with PBS, SP, Curcumin, or SP@Curcumin (n = 3). Scale bar = 50 μm.
